# Sciatica

**Published:** 1903-09-05

**Authors:** 


### SCIATICA.

The existence of sciatica, taking that term to mean a definite painful neuritis of the sciatic nerve, has been called into question more than once of recent years, and there has been a growing tendency to make such a diagnosis only after the most careful process of exclusion, so that by many medical men the term sciatica has come to be looked upon rather as a last resort worthy to be classed with such so-called diagnoses as neuralgia, bilious attack, congestion of the liver, and so forth, which after all are little more than cloaks for professional ignorance used in order to deceive the patient into a belief that his case is perfectly well understood. It is therefore interesting to read of a suggestion which is put forward by Dr. Bruce[1] to the effect that so-called cases of sciatica are simply the early stages of rheumatoid arthritis of the hip-joint. His attention was first directed to the hip-joint as a possible source of the pain by the fact that rest was such an important element in the successful treatment; and at about the same time he came across some remarks by Mr. Jonathan Hutchinson to the effect that some cases of sciatica were really due to trouble in the hip-joint. The nerve-supply of the hip-joint is such as to permit the possibility of pain, when an arthritis is present, being referred to the distribution of the sciatic nerve, as well as to the knee. In 125 specially examined cases of sciatica Dr. Bruce observed gout or rheumatism in 52 per cent., tenderness over the joint capsule in 55 per cent., and wasting of the glutei in 30 per cent., while pain on movement and lameness were common symptoms. A small number of cases which had commenced as sciatica had subsequently developed definite affection of the hip-joint. Swelling of the joint and increased heat in its neighbourhood were seldom observed, owing no doubt to the deep situation of the joint and to the chronicity of the affection. On the whole Dr. Bruce must be given the credit of making a strong case in favour of his theory of the cause of so called sciatica.

[1] Lancet, Aug. 22.

